# Prospective Evaluation of Antibody Response post COVID-19 vaccination in older persons ≧ 60 years old (PEARL 60): A longitudinal 15-months study in a tertiary centre in Malaysia

**DOI:** 10.1371/journal.pone.0340891

**Published:** 2026-02-10

**Authors:** Kejal Hasmukharay, Ashutosh Kumar Verma, Kiirtaara Aravindhan, Prakhaash Raaj S. Alagappan, Aisya Karynna Md Kamal, Nurul Syuhada Zulhaimi, Nor Izzati Saedon, Jamal I-Ching-Sam, Tan Maw Pin, Reena Rajasuriar

**Affiliations:** 1 Division of Geriatric Medicine, Department of Medicine, Faculty of Medicine, Universiti Malaya, Kuala Lumpur, Malaysia; 2 School of Pharmacy, Management & Science University, Kuala Lumpur, Malaysia; 3 Lee Kong Chian School of Medicine, Nanyang Technological University, Singapore, Singapore; 4 Hospital Sibu, Ministry of Health, Kuala Lumpur, Malaysia; 5 Faculty of Medicine, Monash University, Kuala Lumpur, Malaysia; 6 Immunotherapeutics Laboratory (ITL), Faculty of Medicine, Universiti Malaya, Kuala Lumpur, Malaysia; 7 Department of Medical Microbiology, Faculty of Medicine, Universiti Malaya, Kuala Lumpur, Malaysia; 8 Department of Medicine, Faculty of Medicine, Universiti Malaya, Kuala Lumpur, Malaysia; UniCamillus: Saint Camillus International University of Health and Medical Sciences, ITALY

## Abstract

Older adults exhibit heterogeneous immune responses to COVID-19 vaccination, yet the relative contributions of age, comorbidity, vaccine platform, and infection history to antibody durability remain incompletely defined. Understanding these determinants is essential to inform booster strategies in ageing populations. We conducted a longitudinal observational study of 300 participants (250 aged ≥60 years and 50 younger controls) followed for up to 15 months. Anti-spike (anti-S) antibody responses were assessed at four event-anchored timepoints: ≤ 3 months post-primary vaccination (TP1), ~ 3 months post-first booster (TP2), 6–9 months post-first booster capturing waning immunity (TP3), and ≤3 months post-second booster where available (TP4). Multivariable log-linear regression models were used to identify independent determinants of antibody levels, with additional analyses stratified by infection status and vaccine platform. Among older adults, 78.8% had moderate-to-severe comorbidity burden, 40.0% were pre-frail, and only 16.8% received a second booster. At TP3, older age was associated with lower antibody levels in univariable analysis (GMR 0.81, 95% CI 0.68–0.97) but not after adjustment (aGMR 0.78, 95% CI 0.51–1.22, p = 0.279). Independent predictors of higher TP3 antibody levels included female sex (aGMR 1.24, 95% CI 1.02–1.51, p = 0.028), prior SARS-CoV-2 infection (aGMR 1.39, 95% CI 1.14–1.71, p = 0.001), and mRNA (aGMR 5.16, 95% CI 3.57–7.47, p=<0.001) or viral vector boosters (aGMR 6.05, 95% CI 4.03–9.08, p=<0.001), while renal disease was associated with lower responses (aGMR 0.71, 95% CI 0.54–0.94, p = 0.017). Similar associations were observed at TP4. Frailty and sarcopenia were not independently associated with antibody levels. Neutralising antibodies against Omicron were absent after primary vaccination and detected in only 25.9% of infection-naïve older adults after the first booster. Sustained humoral immunity following COVID-19 vaccination is driven primarily by vaccine platform and immune history rather than chronological age or geriatric syndromes alone. Waning immunity unmasks vulnerability in older adults, while low uptake of second boosters highlights a critical gap between immunological risk and vaccine utilisation. These findings support targeted, equity-focused booster strategies prioritising highly immunogenic platforms and high-risk older adults.

## Introduction

Globally, there have been over 778 million confirmed cases of the coronavirus disease 2019 (COVID-19), including 7,095,536 deaths, reported to the World Health Organisation [1] and 5,301,147 cases and over 37 351 deaths in Malaysia by the beginning of May 2025 [2] with recent slight surges in cases, deemed as the periodic COVID-19 waves that are expected throughout the year. The emergence of the COVID-19 pandemic highlighted the vulnerability of older individuals to severe illness and mortality with transmissible respiratory disease. Mortality from COVID appears to increase exponentially after 50 years of age, and most fatalities occurred in those aged 80 years and over [3,4]. Infection with the severe adult respiratory syndrome coronavirus-2 (SARS-CoV-2), however, may present with the full spectrum of symptoms ranging from asymptomatic to catastrophic illness and death in every age group including the oldest old. This variability in health status and outcomes may be understood through a lens of frailty, which is a state of increased vulnerability to adverse health outcomes [5].

Immunity to SARS-CoV-2 induced either through natural infection or vaccination provides protection against reinfection and reduces the risk of clinically significant outcomes [6]. Seropositive recovered subjects have been estimated to have 89% protection from reinfections, and vaccine efficacies from 50 to 95% have been reported [7].In Malaysia, a total of 224.4 vaccine doses has been administered per 100 population, with 85.1 vaccine-persons with complete primary series per 100 population but only 50.5 vaccine-persons with at least one booster or additional dose per 100 population [1].Immune responses tend to wane and become dysregulated with age, through processes known as immunosenescence [5]. Notably, immunosenescence does not occur uniformly across all older adults as they age. Indeed, this variability is hypothesized to be a contributor to frailty itself [8]. Frailty has been correlated with decreased effectiveness of influenza, varicella-zoster, and pneumococcal pneumonia vaccines [9–12].

While vaccination against SARS-CoV-2 has become a crucial strategy to protect high-risk populations, heterogenicity exists in age-related immunogenicity [13–15]. The reduction in death post-COVID-19 hospitalisations after vaccinations is 22.5% lower compared to unvaccinated adults aged 80 years and over [16]. Neutralising antibodies titres are predictive of protection against severe infection [17]. Reduced neutralising activities against emerging variants of concern (VOC) has been recognised in older adults [18]. The duration of protective immunity is also presently unclear. Primary immune responses inevitably wane, with ongoing transmission of increasingly concerning viral variants that may escape control by both vaccine-induced and convalescent immune responses [6]. A critical challenge is to identify the immune correlate (s) of protection from SARS-CoV-2 vaccines in older persons and predict how these guides decisions for future booster doses or even other annual vaccines like influenza.

This study aims to determine the antibody response following the primary series and booster doses of SARSCoV-2 vaccines, measured with a quantitative antibody assay (Roche Elecsys SARS-CoV-2 S) dependent on the different vaccines used. A differential impact of age, frailty, comorbidities and sarcopenia on the antibody response as well as neutralising abilities is also reported here. These findings will help establish the immune responses of older persons to the COVID-19 vaccine in Malaysia, potentially informing future policies on the National Immunization Programme for older persons.

## Methods

### Study approval

The research activities in this study, Prospective Evaluation of Antibody Response Post-COVID-19 Vaccination in Older Persons ≧ 60 years (PEARL 60), were implemented under conditions of written informed consent with protocols approved by the medical ethics committee at the Universiti Malaya Medical Centre (UMMC; no. 202195−10559). The written informed consent permitted storage and de-identified data sharing for research.

### Study design and participants

This study was initiated at the peak of Malaysia’s second wave of the COVID-19 pandemic.

Cases were conveniently recruited from the hospital’s phlebotomy clinic if they met the inclusion criteria stated below from 19^th^ September 2021, while controls were healthy caregivers of cases or medical students at the UMMC. Recruitment ended on 30^th^ September 2021 once a total of 300 participants (cases and controls) were recruited. Participants were initially followed up for 12 months (October 2021-September 2022) but this was extended to 18^th^ December 2022 as soon as second boosters were offered to complete a 15-months follow-up.

Blood sampling was anchored to the most recent antigenic exposure, defined as either completion of the primary vaccine series or receipt of a booster dose. Unless participants were able to give the date of their symptomatic SARS-CoV-2 infection, we used anti-N positivity to suggest a naturally-acquired infection. Four timepoints (TP) were designated: TP1 (≤3 months after completion of the primary vaccine series – captures early post primary response), TP2 (approximately 3 months after the first booster – allows direct comparison with TP1), TP3 (6–9 months after the first booster – captures waning immunity), and TP4 (≤3 months after the second booster, where available – captures sustained immunity due to booster). By using this event-anchored approach rather than fixed calendar months, we accounted for variability in vaccine platform, rollout schedules, and infection events during the study period. For all analyses, results were further stratified by vaccine platform (mRNA, viral vector, inactivated) and infection status (infection-naïve vs previously infected, determined by anti-N serology and clinical history.

### Inclusion and exclusion criteria

For cases, the inclusion criteria were age ≧ 60 years, attending medical subspecialty clinics (geriatrics, cardiology, neurology and nephrology) and primary care clinics after completion of their primary series at recruitment with one and/ or more comorbidities; namely diabetes mellitus, ischemic heart disease, stroke, chronic kidney disease and asthma. Controls are younger, healthy adults with no known comorbidities and completion of primary vaccine series at recruitment.

The exclusion criteria were any active cancer (untreated or undergoing chemotherapy or immunotherapy) and any immunosuppressive drugs taken for non-cancerous conditions.

In this study, younger, healthy adults were selected to serve as a reference group representing the baseline or optimal immune response. Younger, healthy adults have a robust and well-regulated immune system, free from the confounding effects of age-related immune decline.

Using an online sample size calculator by Cleveland Clinic [19], at least total 136 samples; 113 cases and 23 controls will be required to have a confidence level of 95%, powered at 0.8, with probability of having frail cases with immunosenescence of about 20%. Taking into account possible large dropout rates with Movement Control Orders (MCO) implemented nationally, the study team had recruited 250 cases and 50 healthy participants as healthy controls.

### Patient data

A standardised data collection form was used to extract relevant clinical information from the electronic medical records.

Data on demography, comorbidities, Charlson’s Comorbidity Index (CCI), vaccination status, dates and types of vaccines received, dates and types of boosters received, prior COVID-19 infection, frailty using the FRAIL scale, and sarcopenia using the SARC-F scale were collected.

The FRAIL scale [20], a 5-item self-reported screening instrument for frailty, has been identified as practical for use in identifying frailty in the general practice setting. The FRAIL scale has demonstrated preliminary evidence in favour of its predictive validity for mortality. The score ranges from 0–5, with scores of 0 suggesting robust, 1–2 suggesting pre-frail, and ≧3 suggesting frail (S1 Table in Supplementary).

The SARC-F questionnaire has been developed as a rapid diagnostic test for sarcopenia [21]. The self-reported SARC-F components include strength, assistance with walking, rising from a chair, climbing stairs and falls. The scores range from 0 to 10, with 0–2 points for each component and a score of ≧4 suggesting sarcopenia (S2 Table in Supplementary).

### Laboratory assays

Table 1 below summarises the assays used.

**Table 1:** List of different assays used.

Assay Name	Analyzer/ Kit	Detection Target	Units/ Index	Reactive Cut-off	Purpose	Usage in Study
Elecsys Anti-SARS-CoV-2 S	COBAS 8000 (Roche®, Germany)	Spike (S) protein antibodies	U/mL (BAU/mL equivalent)	≥ 0.80 U/mL	Quantify anti-S antibodies from vaccines	All samples in all 4 time points
Elecsys Anti-SARS-CoV-2 N	COBAS 8000 (Roche®, Germany)	Nucleocapsid (N) protein antibodies	COI (Cut-off Index)	≥ 1.0 COI	Detect prior natural infection	All samples in 2nd, 3^rd^ and 4^th^ timepoints
GenScript cPass sVNT	GenScript cPass (L00847)	Neutralizing antibodies blocking RBD-hACE2	% Signal Inhibition	> 30% inhibition	Assess neutralizing capacity (wild type, Delta, Omicron strains) (S1 Fig in Appendix)	68 randomly selected samples amongst the 250 cases, which were available for both the time points 1 and 3.

### Statistical analysis

Statistical analyses were performed using the IBM SPSS Statistics Version 26 (SPSS, Chicago, IL, USA). Frequencies were compared between groups (younger vs older persons) using the χ^2^ test with unpaired, non-parametric Kruskal–Wallis test (two-tailed) for continuous variables and Chi-squared or Fisher’s exact test (sample size < 5) test for categorical variables. Comorbidities were regrouped to cardiometabolic: diabetes, hypertension, ischemic heart disease; renal: chronic kidney disease; neurological: stroke, cognitive impairment; and respiratory: COPD/asthma; malignancy).

As many of the subjects had missing information due to dropouts in timepoint 2 onwards, a method of ‘last observation carry forward’ was applied and results shown used the imputed results. Linear regression analyses were performed to determine the relationship between anti-S results and the possible explanatory variables. Univariable linear regression was first executed to determine the relationship between each of the explanatory variables and anti-S result at timepoint 3 and 4 and by age group (young and old). Any explanatory variable with p-value±<0.20 was included in a stepwise regression. Stepwise regression was used as a step-by-step iterative construction of a regression model that involves the selection of independent variables to be used in a final model. Multivariable linear regression was then performed on all the selected explanatory variables to predict the outcome of anti-S results by each age group. Also, because natural infection can inflate antibody responses, we performed a prespecified sensitivity analysis that excluded participants who seroconverted between TP1 (≤3 months post-primary series) and TP3 (6–9 months post-first booster). The primary model used last observation carried forward (LOCF) for missing follow-ups; the sensitivity analysis used observed data only.

## Results

**Fig 1:**
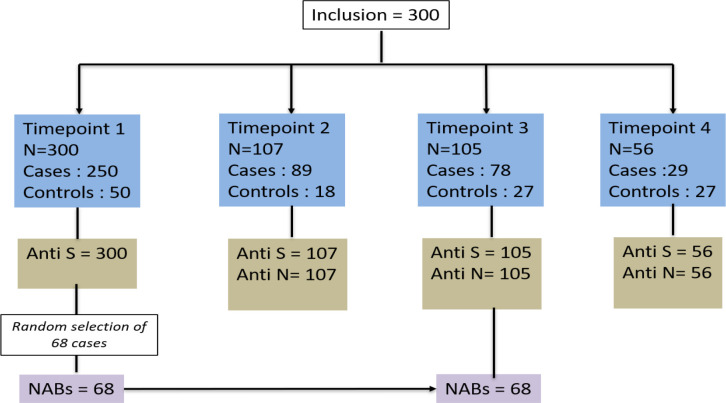
Flowchart demonstrating follow-up rates for each timepoint as well as availability of Anti-S, Anti-N and neutralising antibodies (NABs) during the timepoints.

Data was available for 300 participants with mean age (standard deviation) of 62.4 (16.1) years, and female (51%). Fig 1 depicts the sample selection and breakdown of participants over four time points. Eighty four participants (28%) had at least two follow-ups and 17 participants (5.7%) were followed-up across all four time points (Fig 1).

Amongst older persons (cases), only 16.8% of older persons received their second booster shots, 78.8% of older persons had moderate-severe CCI with cardiometabolic disease – hypertension and diabetes being the commonest comorbidities and 40.0% of older persons were pre-frail (Table 2).

**Table 2:** Comparison of participant characteristics between young control and older persons.

Variables	Controls (n = 50) age < 59 [n (%)]	Cases (n = 250) age ≧ 60 [n (%)]	P value
**Gender**			
Male	15 (30.0)	132 (52.8)	0.003^a^
Female	35 (70.0)	118 (47.2)	
**Comorbidity**			
Cardiometabolic	0	212 (84.8)	<0.001^b^
Renal	0	38 (15.2)	0.001^b^
Neurological	0	21 (8.4)	0.031 ^b^
Respiratory	0	14 (5.6)	0.137^b^
Malignancy	0	14 (5.6)	0.137^b^
Others	0	91 (36.4)	<0.001^b^
**CCI**			
Normal	50 (100)	0	<0.001^b^
Mild	0	53 (21.2)	
Moderate	0	112 (44.8)	
Severe		85 (34.0)	
**Frailty**			
Robust	50 (100)	123 (49.2)	<0.001^b^
Prefrail	0	100 (40.0)	
Frail	0	27 (10.8)	
**Sarcopenia**			
Normal	50 (100)	229 (91.6)	0.034^a^
Sarcopenic	0	21 (8.4)	
**Hx of COVID-19 infection**			
Natural infection (self-reported and confirmed by anti-N)	22 (44.0)	36 (14.4)	<0.001^a^
Asymptomatic natural infection (anti-N positive, no reported infection)	7 (14.0)	43 (17.2)	

^a^ Chi-Square test. All expected counts are > 5.

^b^ Fisher Exact test.

Vaccination platform differed significantly between younger (controls) and older (cases) participants for both the primary vaccination series and the first booster dose, with younger participants more likely to have received mRNA vaccines and older participants more frequently receiving viral vector or inactivated vaccines (Table 3). Patterns of homologous versus heterologous boosting did not differ after the first booster but differed significantly after the second booster, with heterologous schedules more common among older participants. Median time from last antigenic exposure differed between age groups at TP1 and TP3 but not at TP2 or TP4 (Table 3).

**Table 3:** Comparison of types of vaccines received, booster uptake, and median time intervals between time points.

Vaccination Details			
Primary Series Vaccination	Controls	Cases	P Value
mRNA	26 (52.0)	52 (20.8)	<0.001^a^
Viral Vector	10 (20.0)	129 (51.6)	
Inactivated	14 (28.0)	69 (27.6)	
**1**^**st**^ **Booster**			
mRNA	45 (90.0)	159 (63.6)	0.004^a^
Viral Vector	3 (6.0)	55 (22.0)	
Inactivated	1 (2.0)	17 (6.8)	
**1st Booster**			
Homozygous	27 (54.0)	104 (41.6)	0.199^a^
Heterozygous	22 (44.0)	127 (50.8)	
**2**^**nd**^ **Booster**			
mRNA	10 (20.0)	38 (15.2)	1.000^b^
Viral Vector	0	2 (0.8)	
Inactivated	1 (2.0)	3 (1.2)	
**2**^**nd**^ **Booster**			
Homozygous	8 (16.0)	8 (3.2)	0.001^b^
Heterozygous	3 (6.0)	35 (14.0)	
**Median Days (IQR) from Last Antigenic Exposure**			
TP1	99 (78, 112)	72 (54, 91)	<0.001*
TP2	93 (86, 100)	97 (90, 114.5)	0.192*
TP3	171 (159, 183.5)	182.5 (166, 206)	0.026*
TP4	298 (109, 311)	269.5 (202, 287)	0.201*

Table 4 shows antibody responses by vaccination stage, infection status, and vaccine platforms. At TP1 (post-primary vaccination), antibody concentrations varied by vaccine platform and infection status. Among infection-naïve participants, mRNA vaccines and viral vectors elicited higher GMCs compared with inactivated vaccines. Participants with prior infection demonstrated higher GMCs across all vaccine platforms. Following the first booster dose (TP2), GMCs increased substantially across all vaccine platforms and infection strata. Differences between vaccine platforms were less pronounced than at TP1, with overlapping confidence intervals observed for mRNA and viral vector vaccines. At TP3, antibody concentrations reached the upper limit of quantification (250 U/mL) in several strata, particularly among previously infected participants and those receiving mRNA vaccines. By TP4 (post–second booster), GMCs remained at or near the assay upper limit across most vaccine platforms and infection statuses, indicating sustained high antibody concentrations. Across all time points, no participants had antibody levels below the seronegativity threshold (<0.8 U/mL)

**Table 4:** Geometric mean concentrations of anti–S antibodies by vaccination stage, infection status, and vaccine platforms across all the four timepoints.

Vaccination Stage	Infection Status	Primary Vaccine Platform	N	GMC (95% CI)	% < 0.8 U/ml
TP1 (n = 300)	Infection-naive	mRNA	51	187.412 (154.146, 228.005)	0
		Viral Vector	93	194.654 (174.465, 217.179)	0
		Inactivated	48	28.516 (19.719, 41.237)	0
	Past-infection	mRNA	27	211.265 (160.341, 278.364)	0
		Viral Vector	46	206.827 (183.313, 233.358)	0
		Inactivated	35	49.947 (31.893, 78.222)	0
TP2 (n = 107)	Infection-naive	mRNA	16	250.000 (250.000, 250.000)	0
		Viral Vector	27	233.934 (213.111, 256.791)	0
		Inactivated	14	142.381 (64.624, 313.697)	0
	Past-infection	mRNA	10	237.550 (211.626, 266.650)	0
		Viral Vector	25	222.902 (175.905, 282.455)	0
		Inactivated	15	245.489 (237.568, 253.674)	0
TP3 (n = 105)	Infection-naive	mRNA	11	250.000 (250.000, 250.000)	0
		Viral Vector	25	211.727 (174.694, 256.611)	0
		Inactivated	8	243.426 (228.560, 259.259)	0
	Past-infection	mRNA	16	250.000 (250.000, 250.000)	0
		Viral Vector	23	250.000 (250.000, 250.000)	0
		Inactivated	22	250.000 (250.000, 250.000)	0
TP4 (n = 56)	Infection-naive	mRNA	4	250.000 (250.000, 250.000)	0
		Viral Vector	11	243.606 (229.940, 258.083)	0
		Inactivated	4	169.237 (48.893, 585.794)	0
	Past-infection	mRNA	13	250.000 (250.000, 250.000)	0
		Viral Vector	14	250.000 (250.000, 250.000)	0
		Inactivated	10	250.000 (250.000, 250.000)	0

i. GMCs computed on log-transformed anti-S (Roche Elecsys) and back-transformed; 95% CIs from log-scale SE.

ii. Infection status defined by anti-N serology and/or recorded PCR/antigen test; “infection-naïve” = no evidence pre-TP.

iii. % < 0.8 U/mL shown for biological context (seronegativity threshold).

iv. Timepoints anchored to antigenic event (TP1 = post-primary; TP2/TP3 = post-1st booster; TP4 = post-2nd booster).

v. N reflects non-missing titres at each stratum.

In the sub study, involving only 68 persons from the cases (older persons), neutralising antibody activity varied substantially by viral strain, vaccination stage, and prior infection status. Against the wild-type and Delta strains, a high proportion of participants achieved ≥30% inhibition following the first booster (TP3), with near-universal neutralisation observed among both infection-naïve and previously infected individuals across mRNA and viral vector vaccines. In contrast, neutralising activity against the Omicron variant was markedly reduced after primary vaccination, with no participants achieving ≥30% inhibition at TP1, regardless of infection status or vaccine platform. Following booster vaccination, Omicron-specific neutralisation improved, particularly among previously infected participants, though responses remained lower and more variable compared with wild-type and Delta strains (Table 5).

**Table 5:** Neutralising antibodies (NABs) results against SARS-CoV-2 wild-type, Delta, and Omicron strains by vaccination stage, infection status, and vaccine platforms.

Strain	Time Point	Infection Status	Vaccine Platform Booster	N = 68	n ≥ 30% (%)	95% CI	Median % Inhibition (IQR)
Wild Type	TP1 (≤3 mo post-primary)	Infection-naive	mRNA	27	22 (81.48)	58.97, 78.96	75.16 (51.2-88.64)
			Vector vaccine	6	6 (100)	36.39, 78.38	55.22 (39.9-66.36)
			Inactivated	0	0	–	–
		Past-infection	mRNA	24	18 (75.0)	57.35, 78.88	60.84 (51.57-93.06)
			Vector vaccine	7	7 (100)	54.84, 95.43	80.97 (62.54-92.96)
			Inactivated	4	1 (25.0)	–	58.98 (58.98)
	TP3 (6–9 months post 1st booster)	Infection-naive	mRNA	27	27 (100)	91.57, 97.20	97.41 (94.1-97.71)
			Vector vaccine	6	5 (83.33)	45.25, 93.52	77.42 (68.12-81.61)
			Inactivated	0	0	–	–
		Past-infection	mRNA	24	24 (100)	94.67, 97.95	97.62 (97.01-97.76)
			Vector vaccine	7	7 (100)	75.54, 105.50	97.68 (91.99-97.86)
			Inactivated	4	4 (100)	61.32, 115.24	96.16 (78.83-97.74)
Delta	TP1 (≤3 mo post-primary)	Infection-naive	mRNA	27	17 (62.96)	53.86, 75.85	61.29 (52.16-82.81)
			Vector vaccine	6	3 (50.0)	−16.56, 121.11	40.17 (32.68-83.98)
			Inactivated	0	0	–	–
		Past-infection	mRNA	24	16 (66.67)	45.71, 71.42	45.98 (37.62-83.05)
			Vector vaccine	7	6 (85.71)	46.20, 84.94	66.61 (45.46-82.06)
			Inactivated	4	1 (25.00	–	44.6 (44.6)
	TP3 (6–9 months post 1st booster)	Infection-naive	mRNA	27	27 (100)	81.80, 94.57	96.03 (83.26-97.02)
			Vector vaccine	6	4 (66.67)	30.32, 80.67	58.99 (44.7-66.29)
			Inactivated	0	0	–	–
		Past-infection	mRNA	24	24 (100)	87.82, 97.92	97.25 (96.59-97.48)
			Vector vaccine	7	7 (100)	60.17, 104.4	97.22 (71.69-97.343)
			Inactivated	4	4 (100)	43.62, 120.01	91.50 (66.49-97.14)
Omicron	TP1 (≤3 mo post-primary)	Infection-naive	mRNA	27	0	–	–
			Vector vaccine	6	0	–	–
			Inactivated	0	0	–	–
		Past-infection	mRNA	24	0	–	–
			Vector vaccine	7	0	–	–
			Inactivated	4	0	–	–
	TP3 (6–9 months post 1st booster)	Infection-naive	mRNA	27	7 (25.93)	39.10, 69.65	57.98 (32.57-69.19)
			Vector vaccine	6	0	–	–
			Inactivated	0	0	–	–
		Past-infection	mRNA	24	18 (75.0)	72.84, 88.66	84.03 (66.24-95.73)
			Vector vaccine	7	4 (57.14)	80.77, 96.08	90.44 (85.7-91.15)
			Inactivated	4	2 (50.0)	−185.12, 333.54	74.21 (53.8-94.62)

In univariable analyses, older age was associated with lower antibody levels at TP3 compared with younger participants (GMR 0.814, 95% CI 0.681–0.974; p = 0.025), although this association was attenuated and no longer significant after multivariable adjustment (aGMR 0.784, 95% CI 0.505–1.219; p = 0.279). Female sex remained independently associated with higher TP3 antibody levels compared with male sex (aGMR 1.242, 95% CI 1.024–1.507; p = 0.028). Renal comorbidity was independently associated with lower antibody levels (aGMR 0.712, 95% CI 0.537–0.941; p = 0.017), whereas cardiometabolic and neurological comorbidities were not significantly associated with TP3 antibody levels in the adjusted model. Prior SARS-CoV-2 infection was independently associated with higher antibody levels at TP3 (aGMR 1.394, 95% CI 1.138–1.708; p = 0.001). Vaccine platform for the first booster showed the strongest association with TP3 responses: compared with an inactivated booster, mRNA (aGMR 5.163, 95% CI 3.571–7.466; p < 0.001) and viral vector boosters (aGMR 6.045, 95% CI 4.027–9.076; p < 0.001) were associated with substantially higher antibody levels. Frailty category and sarcopenia were not independently associated with TP3 antibody levels (Table 6).

**Table 6:** Univariable and multivariable predictors of anti-spike antibody levels at 6–9 months after first booster vaccination (TP3).

Covariate	Levels	Univariable GMR at TP3 (95% CI)	P Value	Multiivariable aGMR at TP3 (95% CI)	P Value
Age	Young	1		1	
	Old	0.814 (0.681, 0.974)	0.025	0.784 (0.505, 1.219)	0.279
Sex	Male	1		1	
	Female	1.227 (1.011, 1.489)	0.038	1.242 (1.024, 1.507)	0.028
Cardiometabolic	No	1		1	
	Yes	0.880 (0.724, 1.071)	0.202	0.923 (0.705, 1.209)	0.560
Renal	No	1		1	
	Yes	0.781 (0.533, 1.146)	0.206	0.712 (0.537, 0.941)	0.017
Neurological	No	1		1	
	Yes	1.139 (0.886, 1.464)	0.309	1.222 (0.829, 1.802)	0.310
Infection status	Infection-naïve	1		1	
	Past infection	1.360 (1.138, 1.626)	0.001	1.394 (1.138, 1.708)	0.001
Vaccine Platform (1^st^ Booster)	Inactivated	1		1	
	mRNA	4.751 (2.156, 10.470)	<0.001	5.163 (3.571, 7.466)	<0.001
	Viral vector	5.156 (2.327, 11.423)	<0.001	6.045 (4.027, 9.076)	<0.001
Frailty	Robust	1		1	
	Pre Frail	1.007 (0.814, 1.246)	0.951	1.074 (0.867, 1.329)	0.512
	Frail	1.132 (0.895, 1.432)	0.300	1.189 (0.782, 1.809)	0.416
Sarcopenia	No	1		1	
	Yes	1.010 (0.716, 1.425)	0.954	0.797 (0.525, 1.210)	0.286

i. Values are presented as geometric mean ratios (GMR) from univariable analyses and adjusted geometric mean ratios (aGMR) from multivariable models, with 95% confidence intervals.

ii. TP3 corresponds to measurements obtained 6–9 months after the first booster vaccination, representing the period of waning humoral immunity.

In univariable analyses at TP4, older age was associated with lower anti-spike antibody levels, although this association was attenuated after multivariable adjustment. Female sex, renal comorbidity, prior SARS-CoV-2 infection, and first-booster vaccine platform remained independently associated with antibody levels at TP4, while cardiometabolic and neurological comorbidities, frailty status, and sarcopenia were not. Together with the TP3 findings, these results indicate that host and vaccine-related factors continue to influence antibody levels across booster timepoints, with consistent effects observed for sex, renal comorbidity, prior infection, and booster platform (Table 7).

A sensitivity analysis excluding participants with evidence of intercurrent SARS-CoV-2 infection between TP1 and TP3 (n = 12) was performed, leaving 93 participants with observed TP3 data. In this restricted cohort, most associations observed in the primary analysis were attenuated after multivariable adjustment, with cardiometabolic comorbidity remaining independently associated with higher antibody levels (adjusted GMR 1.009, 95% CI 1.002–1.016). Similar analysis was not done for TP4 given the small number. (S3 Table in supplementary).

**Table 7:** Univariable and multivariable predictors of anti-spike antibody levels at 3 months after second booster vaccination (TP4).

Covariate	Levels/ Comparison	Univariable GMR at TP4 (95% CI)	P Value	Multivariable aGMR at TP4 (95% CI)	P Value
Age	Young	1		1	
	Old	0.788 (0.666, 0.933)	0.006	0.767 (0.493, 1.193)	0.238
Sex	Male	1		1	
	Female	1.245 (1.028, 1.509)	0.025	1.224 (1.008, 1.486)	0.041
Cardiometabolic	No	1		1	
	Yes	0.878 (0.722 = 3, 1.067)	0.192	0.940 (0.716, 1.232)	0.651
Renal	No	1		1	
	Yes	0.773 (0.527, 1.134)	0.187	0.709 (0.536, 0.938)	0.016
Neurological	No	1		1	
	Yes	1.128 (0.878, 1.449)	0.346	1.222 (0.828, 1.801)	0.311
Infection status	Infection-naïve	1		1	
	Past infection	1.373 (1.151, 1.637)	<0.001	1.368 (1.5, 1.680)	0.003
Vaccine Platform (1st Booster)	Inactivated	1		1	
	mRNA	4.599 (2.056, 10.291)		5.318 (3.626, 7.799)	<0.001
	Viral vector	4.948 (2.200, 11.131)	<0.001	6.263 (4.117, 9.526)	<0.001
Frailty	Robust	1		1	
	Pre-Frail	0.980 (0.792, 1.213)	0.853	1.067 (0.861, 1.321)	0.553
	Frail	1.110 (0.878, 1.403)	0.382	1.216 (0.799, 1.850)	0.361
Sarcopenia	No	1		1	
	Yes	1.000 (0.709, 1.411)	0.998	0.799 (0.523, 1.203)	0.274

**Fig 2:**
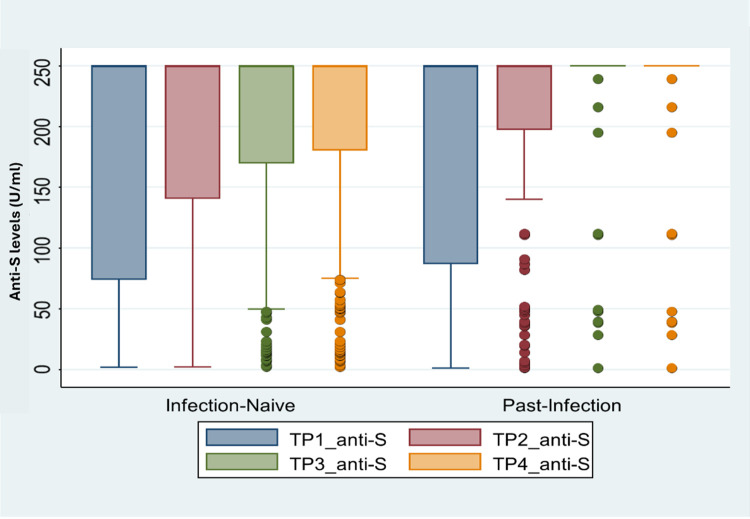
Anti-S antibody levels acrossTP1-TP4 stratified by SARS-CoV-2 infection status.

Fig 2 demonstrates longitudinal anti-spike (anti-S) antibody levels across four timepoints stratified by infection status (infection-naïve vs past infection). Across all timepoints, participants with prior SARS-CoV-2 infection consistently exhibit higher anti-S antibody titres compared with infection-naïve individuals. This separation is most evident at later timepoints, particularly during the waning phase after the first booster (TP3) and following subsequent boosting (TP4), where antibody levels in the past-infection group remain clustered at higher values with less dispersion. In contrast, infection-naïve participants show greater variability and lower median antibody levels over time, with more pronounced spread and lower values evident during later timepoints (Fig 2).

## Discussion

In this longitudinal observational study, we evaluated determinants of anti-S antibody levels across multiple post-vaccination timepoints encompassing both waning immunity after the first booster and early responses following a second booster. Multivariable analyses consistently identified booster vaccine platform, prior SARS-CoV-2 infection, sex, and renal comorbidity as independent correlates of antibody magnitude at both TP3 and TP4, whereas chronological age, frailty status, sarcopenia, cardiometabolic disease, and neurological comorbidity were not independently associated after adjustment. Longitudinal visualisation of antibody distributions corroborated these findings, demonstrating persistently higher antibody levels among previously infected individuals compared with infection-naïve participants, particularly at later timepoints. Collectively, these results indicate that heterogeneity in vaccine-induced humoral responses is largely explained by vaccine- and exposure-related factors rather than chronological age or global vulnerability measures alone, underscoring the importance of considering immune history and specific comorbid conditions when interpreting post-vaccination antibody responses in older adults.

The most striking finding in this study was the dominant influence of booster vaccine platform on antibody levels. Participants primed with inactivated vaccines had recorded lower aGMR than their mRNA-or-virus vector-primed peers, an observation that mirrors the phase II data in Chinese adults where the inactivated vaccines elicited substantially weaker neutralisation than the mRNA vaccine [22]. Compared with inactivated vaccines, both mRNA and viral vector boosters were associated with more than fivefold higher antibody titres. This observation is consistent with randomised trials and real-world studies demonstrating superior immunogenicity of mRNA and viral vector boosters following inactivated primary series, mediated through more efficient spike protein expression, robust germinal centre responses, and enhanced memory B-cell induction [23,24]. Various other head-to-head studies corroborate this effect observed in our study. In Hong Kong, comparison of 366 adults with 82 participants aged >60 year years showed geometric mean neutralising antibody titre was 3.9-fold higher with the mRNA vaccine (BNT162b2) than with an inactivated vaccine [25]. Similarly, a parallel study quantified higher median spike-binding IgG after mRNA (BNT162b2) vaccine (473 AU mL ⁻ ¹) compared inactivated vaccine (124 AU mL ⁻ ¹) after adjusting for age, sex and comorbidity burden [26]. This disparity reflects the alum-only adjuvant used in inactivated vaccines compared to higher spike expression induced by mRNA vaccines, compounded by immunosenescence in older participants. From a public health perspective, these findings have direct implications for booster policy, particularly in settings where inactivated vaccines were widely deployed during the primary rollout. Prioritising higher-immunogenicity booster platforms may be especially important for older adults and clinically vulnerable groups, in whom maximising durable protection is critical.

Female sex was independently associated with higher antibody levels at TP3, even after adjustment for confounders. This finding aligns with extensive literature demonstrating stronger humoral immune responses in females following vaccination and natural infection [27–30]. Biological mechanisms include the immunomodulatory effects of sex hormones, differential expression of X-linked immune genes, and enhanced B-cell activation and antibody production in females [27,31,32]. While higher antibody titres may translate into improved short-term protection, they are also associated with increased reactogenicity, underscoring the complex trade-offs inherent in sex-specific immune responses. The persistence of this association several months after booster vaccination supports sex as a stable modifier of vaccine-induced immunity rather than a transient early effect [32].

Among comorbidities examined, renal disease emerged as an independent predictor of lower antibody levels. Chronic kidney disease is well recognised to impair both innate and adaptive immune responses through mechanisms including uremia-associated immune dysfunction, impaired antigen presentation, reduced memory B-cell formation, and chronic systemic inflammation [33,34]. Studies have consistently demonstrated reduced serological responses to COVID-19 vaccines in individuals with renal impairment, including those not receiving dialysis [35,36]. In contrast, cardiometabolic and neurological comorbidities were not independently associated with antibody levels after adjustment, suggesting that their effects may be mediated indirectly or outweighed by stronger determinants such as vaccine platform and prior infection. These findings underscore the need for heightened vigilance and potentially tailored booster strategies in individuals with renal disease, who already face disproportionate risks of severe COVID-19 outcomes.

Prior SARS-CoV-2 infection was strongly and independently associated with higher antibody levels across timepoints, consistent with the concept of hybrid immunity. Hybrid immunity occurs when individuals have experienced both natural infection and vaccination, leading to enhanced immune responses compared to either exposure alone. Repeated antigenic exposure through infection followed by vaccination enhances both the magnitude and breadth of humoral responses, including improved neutralisation against divergent variants [37–40]. The persistence of this effect at 6–9 months post-first booster indicates that infection-induced immunological memory remains functionally relevant beyond the early post-vaccination period. Population-based studies, including those focused on older adults, have shown that hybrid immunity confers more durable immune responses than vaccination alone [39]. These evidences has important clinical implications: 1) Increased protection against variants implying that this resilience can greatly inform vaccination strategies, particularly in the context of emerging variants [41], 2) Guiding Vaccination Recommendations and 3) Resource allocation so that healthcare providers can prioritize vaccination efforts and resources within populations [39]. However, this immunological advantage must be interpreted cautiously, as infection-acquired immunity comes at the cost of acute morbidity and potential long-term sequelae, particularly in older and vulnerable populations.

Within three months of receiving the first booster dose, only half of included participants had neutralising antibodies against the Omicron strains. This may suggest evasion of the Omicron variant from spike protein-specific immunity. Several studies had similar findings, demonstrating extensive reduction in neutralising antibodies against the Omicron strains compared to the Wuhan/wild type and delta strains [42–45]. At three months post-primary series, binding titres ≥250 U mL ⁻ ¹ predicted wild-type and Delta neutralisation (p ≤ 0.005), yet offered no protection against Omicron BA.1, a finding echoed by global reports of near-complete immune escape in older adults after two or even three prototype-based doses [46,47].

Alarmingly, only 16.8% of older adults availed themselves of a second booster, mirroring regional data on booster hesitancy despite clear vulnerability to immune-evasive strains. A web-based cross-sectional study with 798 local respondents showed a prevalence of second COVID booster hesitancy to be 26.7% with older age (AOR = 1.040, 95% CI 1.022–1.058), concern about serious long term side effects of the vaccine (AOR = 4.010, 95% CI = 2.218–7.250), and opinions of close friends and immediate family members that the booster is harmful (AOR = 2.201, 95% CI = 1.280–3.785) being the main predictors [48]. A study by Jeffrey et al which included 23 000 adults from 23 countries also reported that COVID-19 vaccine booster acceptance among those vaccinated decreased from 87.9% in 2022 to 71.6% in 2023 (P < 0.001) [48].

Although geriatric syndromes of frailty and sarcopenia and multimorbidity did not attain statistical significance in our adjusted models, this null finding is most plausibly explained by limited power rather than a true lack of biological effect. Only 27 (10.8%) participants met frailty criteria and 21 (8.4%) met sarcopenia criteria, yielding <40% power to detect a between-group difference. Studies consistently highlight frailty as a significant predictor of impaired immune responses to vaccinations, including COVID-19 mRNA vaccines. Kakugawa et al. demonstrate that frailty significantly diminishes vaccine response effectiveness in older adults, emphasizing that this demographic is at an increased risk for suboptimal immunogenicity following vaccination [49]. Similarly, a study by Demaret et al. details impaired functional T-cell responses to the BNT162b2 vaccine in older individuals [50]. While frailty has been linked to impaired vaccine responses and adverse COVID-19 outcomes in these studies, its relationship with humoral immunity appears complex and context-dependent. Booster vaccination may partially mitigate frailty-related disparities in antibody responses, as suggested by studies demonstrating restoration of humoral responses after additional doses [51,52]. Frailty has been associated with decreased antibody after the primary series with the booster vaccination overcoming the effects of COVID-19 infection and frailty on antibody levels, hence suggesting maximal generation of antibodies can be reached with appropriate boosting even in frail older adults [12], though the true underpinning association between frailty and vaccine responsiveness remains poorly defined [43,53–55]. Alternatively, frailty may exert a greater influence on cellular immunity, inflammatory regulation, or the clinical consequences of immune escape rather than on circulating antibody titres alone [56,57]. Limited power, heterogeneity in frailty measurement, and conservative imputation of missing data may also have reduced sensitivity to detect any modest associations in this study. These findings reinforce that antibody titres should not be interpreted as the sole correlate of protection in frail older adults and highlight the importance of multidimensional immune assessment.

Exclusion of participants with intercurrent SARS-CoV-2 infection in the sensitivity resulted in marked attenuation of associations observed in the primary TP3 analysis, highlighting the dominant contribution of infection-related antibody boosting during the waning phase after the first booster. The loss of statistical significance for most covariates is likely attributable to reduced sample size and limited outcome variability rather than absence of underlying biological effects. The persistence of an association with cardiometabolic comorbidity should be interpreted cautiously given these constraints. Collectively, these findings underscore the need to account for intercurrent infection in longitudinal immunogenicity analyses and indicate that comorbidity-related differences are most discernible prior to infection-driven amplification of antibody responses.

Several limitations should be acknowledged. Follow-up was disrupted by COVID-19 movement control orders, resulting in attrition at later timepoints, particularly at TP3 and TP4. Missing data were addressed using last observation carried forward, which assumes stability of antibody levels and may underestimate true waning over time. In addition, only a small proportion of participants received a second booster during the study period, limiting statistical power at TP4 and potentially attenuating adjusted estimates. Antibody titres were used as the primary immunogenicity outcome and may not fully capture cellular or functional immunity, particularly in older or frail individuals. Finally, as an observational study, residual confounding cannot be excluded. These limitations should be considered when interpreting the findings, particularly with respect to temporal comparisons and subgroup analyses. This study has several strengths too. It employed a longitudinal design with clearly defined, event-anchored timepoints capturing early post-vaccination responses, waning immunity, and post-booster restoration. Inclusion of both older and younger adults allowed age-related comparisons, while detailed characterisation of vaccine platforms, infection history, comorbidities, frailty, and sarcopenia enabled multivariable adjustment for key confounders. The use of geometric mean ratios provided an appropriate analytic framework for skewed antibody distributions.

In conclusion, this study provides evidence that heterogeneity in post-vaccination antibody responses among older adults is driven predominantly by vaccine platform, immune history, and specific comorbid conditions rather than chronological age or geriatric syndromes alone. Periods of waning immunity appear to unmask these differences most clearly, while recent booster exposure can partially restore humoral responses and reduce inter-individual variability. However, the low uptake of second booster doses among older adults, coupled with diminished neutralisation against immune-evasive variants, underscores a critical gap between immunological vulnerability and real-world vaccine utilisation. These findings support a shift from age-based vaccination paradigms towards more targeted booster strategies that prioritise high-immunogenicity platforms, timely boosting, and proactive outreach to older adults with high-risk comorbidities. Integrating immunogenicity data with geriatric and population-level considerations will be essential to inform equitable, adaptive vaccination policies as SARS-CoV-2 continues to evolve.

## Supporting information

S1 Table Frailty Assessment Questionnaire (FRAIL SCALE). (PDF)

S2 Table Sarcopenia Screening Tool (SARC-F). (PDF)

S3 Table Sensitivity analysis excluding seroconverters between TP1 and TP3. (PDF)

S1 Fig Trends of Circulating Strains in Malaysia. (PDF)
