# Supplementary material for: Prospective Evaluation of Antibody Response post COVID-19 vaccination in older persons ≧ 60 years old (PEARL 60): A longitudinal 15-months study in a tertiary centre in Malaysia
Source: PLoS One. 2026 Feb 10;21(2):e0340891. doi: 10.1371/journal.pone.0340891 (PMC12890099; doi:10.1371/journal.pone.0340891)
Supplement: S1 Table — (PDF) [file pone.0340891.s001.pdf]

Supporting Information

S1 Table : Frailty Assessment Questionnaire (FRAIL SCALE)

Fatigue: How much of the time during the past 4 weeks did you feel tired?

1 = All of the time, 2 = Most of the time, 3 = Some of the time, 4 = A little of the time, 5 = None of the time. Responses of “1” or “2” are scored as 1 and all others as 0.

Score_____

Resistance: By yourself and not using aids, do you have any difficulty walking up 10 steps without resting? 1 = Yes, 0 = No.

Score_____

Ambulation: By yourself and not using aids, do you have any difficulty walking a couple of blocks (e.g. several hundred yards)? 1 = Yes, 0 = No.

Score_____

Illnesses: Did a doctor ever tell you that you have [illness]? How many (see list below): _____

The total illnesses (0–11) are recoded as 0–4 = 0 and 5–11 = 1.

The illnesses include hypertension, diabetes, cancer (other than a minor skin cancer), chronic lung disease, heart attack, congestive heart failure, angina, asthma, arthritis, stroke, and kidney disease. Score_____

Loss of weight: How much do you weigh? __________[current weight]

One year ago, how much did you weigh?_________[weight 1 year ago]

” Percent weight change is computed as: [[weight 1 year ago - current weight]/weight 1 year ago]] * 100.

Percent change > 5 (representing a 5% loss of weight) is scored as 1 and < 5% as 0.

Score_____

Total Score: _______

A score of 0 represents robust health status, 1-2: Pre-frail 3-5, Frail
