# Supplementary material for: Prospective Evaluation of Antibody Response post COVID-19 vaccination in older persons ≧ 60 years old (PEARL 60): A longitudinal 15-months study in a tertiary centre in Malaysia
Source: PLoS One. 2026 Feb 10;21(2):e0340891. doi: 10.1371/journal.pone.0340891 (PMC12890099; doi:10.1371/journal.pone.0340891)
Supplement: S2 Table — (PDF) [file pone.0340891.s002.pdf]

S2 Table : Sarcopenia Screening Tool (SARC-F)

Component	Question	Scoring	Score
**Strength**	How much difficulty do you have in lifting and carrying 10 pounds?	None = 0<br>Some = 1<br>A lot or unable = 2	
**Assistance in walking**	How much difficulty do you have walking across a room?	None = 0<br>Some = 1<br>A lot, use aids, or unable = 2	
**Rise from a chair**	How much difficulty do you have transferring from a chair or bed?	None =0<br>Some =1<br>A lot or unable without help = 2	
**Climb stairs**	How much difficulty do you have climbing a flight of 10 stairs?	None = 0<br>Some =1<br>A lot or unable = 2	
**Falls**	How many times have you fallen in the past year?	None =0<br>Some = 1<br>A lot or unable= 2	
		**TOTAL SCORE**	

A score of 0-3 = low risk of sarcopenia

A score ≥ 4 = at risk of sarcopenia
