# Supplementary material for: Prospective Evaluation of Antibody Response post COVID-19 vaccination in older persons ≧ 60 years old (PEARL 60): A longitudinal 15-months study in a tertiary centre in Malaysia
Source: PLoS One. 2026 Feb 10;21(2):e0340891. doi: 10.1371/journal.pone.0340891 (PMC12890099; doi:10.1371/journal.pone.0340891)
Supplement: S3 Table — (PDF) [file pone.0340891.s003.pdf]

S3 Table : Sensitivity analysis excluding seroconverters between TP1 and TP3

		Univariable Regression			Multivariable Regression (n=44)	
Covariate	Levels	n	GMR at TP3 (95% CI)	P Value	Multiivariable aGMR at TP3 (95% CI)	P Value
Age	Young	93	1		1	
	Old		0.936 (0.871, 1.006)	0.073	0.992 (0.983, 1.001)	0.078
Sex	Male	93	1		1	
	Female		1.037 (0.933, 1.154)	0.496	1.003 (0.997, 1.009)	0.293
Cardiometabolic	No	93	1		1	
	Yes		1.028 (0.924, 1.143)	0.610	1.009 (1.002, 1.016)	0.013
Renal	No	93	1		1	
	Yes		1.051 (0.995, 1.110)	0.074	1.000 (0.983, 1.017)	0.975
Neurological	No	93	1		1	
	Yes		1.012 (0.930, 1.102)	0.777	1.002 (0.989, 1.015)	0.753
Infection status	Infection-naïve	93	1		1	
	Past infection		1.104 (0.992, 1.229)	0.069	1.005 (0.999, 1.011)	0.078
Vaccine Platform (1st Booster)	Inactivated	93	1		1	
	mRNA		0.976 (0.934, 1.019)	0.266	1.003 (0.991, 1.015)	0.671
	Viral vector		0.864 (0.708, 1.056)	0.151	1.005 (0.991, 1.019)	0.504
Frailty	Robust		1		1	
	Pre Frail		0.978 (0.849, 1.126)	0.751	1.001 (0.995, 1.008)	0.677
	Frail		1.012 (0.931, 1.099)	0.782	1.004 (0.991, 1.016)	0.547
Sarcopenia	No	93	1		1	
	Yes		1.051 (0.995, 1.110)	0.074	0.999 (0.988, 1.011)	0.920

N=12 seroconverters, restricted sample, n=93

Seroconverters = new evidence of infection occurring after TP1 and before/at TP3 as evidenced by Anti-N negative at TP2 and anti-N positive at TP3 and / or documented PCR/antigen-confirmed infection with onset date between TP1 and TP3, if dates were available.
