# Supplementary material for: Prospective Evaluation of Antibody Response post COVID-19 vaccination in older persons ≧ 60 years old (PEARL 60): A longitudinal 15-months study in a tertiary centre in Malaysia
Source: PLoS One. 2026 Feb 10;21(2):e0340891. doi: 10.1371/journal.pone.0340891 (PMC12890099; doi:10.1371/journal.pone.0340891)
Supplement: S1 Fig — (PDF) [file pone.0340891.s004.pdf]

S3 Fig : Trends of Circulating Strains in Malaysia

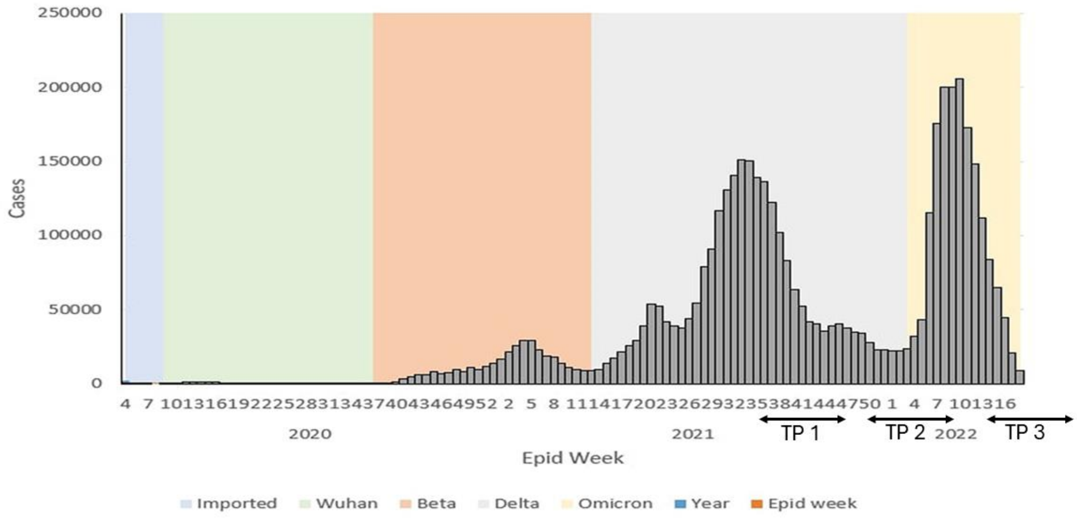

COVID 19 epidemiological curve in Malaysia from the very first case detection till first quarter of 2022, corresponding to the different circulation strains.
